# Assessment of medication review among pharmacy professionals in the UAE: A cross-sectional study on knowledge, attitudes, practices, and barriers

**DOI:** 10.1371/journal.pone.0337233

**Published:** 2025-11-18

**Authors:** Rizah Anwar Assadi, Semira Beshir, Omaimah Toufiq, Selma Benwahmane Castell, Mariam Jihad Diab, Shabaz Mohiuddin Gulam

**Affiliations:** 1 Department of Pharmacy Practice, College of Pharmacy, Dubai Medical University, Dubai, United Arab Emirates; 2 Department of Pharmacy Practice, College of Pharmacy, Gulf Medical University, Ajman, United Arab Emirates,; 3 Clinical Pharmacy Department, Thumbay University Hospital, Ajman, United Arab Emirates; Medicine Information Services Unit, Pharmacy Services Division, ERITREA

## Abstract

**Background:**

Medication reviews are vital for optimizing patient care and resolving drug-related problems, with pharmacists playing a key role. Understanding pharmacists’ knowledge, attitudes, perceived barriers, and practices regarding medication reviews in the UAE is crucial for enhancing medication safety.

**Aim:**

To assess the knowledge, attitudes, perceived barriers, and practices of pharmacy professionals in the UAE concerning medication reviews.

**Methods:**

An online cross-sectional survey was distributed to licensed pharmacists in the UAE with at least a bachelor’s degree. The survey collected data on demographics, knowledge, attitudes, perceived barriers, and practices related to medication reviews. Descriptive statistics, Mann-Whitney U tests, Kruskal-Wallis H tests, and Spearman’s rank correlation were used for data analysis.

**Results:**

Of 426 pharmacists approached, 421 responded (98.8%). Most agreed that medication reviews should be pharmacist-led (99.5%) and include comprehensive patient profiles (99.0%). Awareness was high for prescription reviews (94.3%) but low for Pharmaceutical Care Network Europe classifications (4.3%). Positive attitudes were widespread (98.8%), and major barriers included time constraints (83.6%) and workload (76.7%). Around 73% conducted medication reviews, but over half did not use standardized tools. Gender differences were found in knowledge (p = 0.003) and attitudes (p = 0.007).

**Conclusion:**

Pharmacists in the UAE have positive attitudes toward medication reviews but face barriers, particularly time constraints and workload. Enhanced training is needed to address these issues and improve patient outcomes.

## Introduction

Medication therapy is essential for managing various health conditions, as there is a growing reliance on pharmacotherapy to enhance patient outcomes and quality of life. However, the complexity of medication regimens, particularly for patients with chronic conditions or multiple comorbidities, increases the risk of drug-related problems (DRPs). These problems include adverse drug reactions, medication errors, and issues with nonadherence. Consequently, DRPs can result in hospitalizations, higher healthcare costs, and less-than-optimal therapeutic outcomes [1–3]. DRPs encompass issues related to drug selection, dosing, and prescribing errors, including inappropriate or unnecessary medications, potential drug–drug interactions, and incorrect dosage regimens [4].

Medication Review is vital to ensure that medication use is optimized. According to the Pharmaceutical Care Network Europe (PCNE), Medication Reviews are defined as “a structured evaluation of a patient’s medicines with the aim of optimizing medicines use and improving health outcomes, entailing detecting DRPs and recommending interventions” [5]. There is no standardized classification for medication reviews globally. There are three types of medication reviews according to the Pharmaceutical Care Network Europe (PCNE). These include 1 (Simple), 2A and 2B (Intermediate) and 3 (Advanced). PCNE Type 1 involves a simple medication review on the basis of pharmacy history. PCNE Type 2A is an intermediate review based on patient information and medication history. PCNE Type 2B is also classified as an intermediate review if general practitioner information is available. PCNE Type 3 is an advanced review involving medication history, patient information, and clinical information [4]. Similarly, in the United Kingdom, key ideas for the medication review process are outlined in Room for Review, a document released by the Taskforce on Medicines Partnership in 2002. It places a strong emphasis on documentation, patient agreement on modifications, systematic evaluation by a qualified individual, and impact monitoring. This document is followed by the 2008 Room for Review document, which is complemented with information on different types of medication reviews with examples. Level 0 refers to an unplanned, unstructured review in which the patient may not even be present. Level 1 “prescription review” conducted by a single healthcare provider, typically without the use of complete medical records. Level 2 – “compliance and concordance review” with complete medical notes, which are frequently missing. Level 3, known as “clinical medication review” with medical notes and patient present, the review can be done by single healthcare provider or multidisciplinary team [6]. Pharmacists, with their extensive knowledge of pharmacotherapy and accessibility within the healthcare system, are uniquely qualified to lead these reviews. There are different types of Medication Review: unplanned reviews conducted without the patient’s presence, prescription reviews performed by a single provider, compliance reviews utilizing complete medical records, and clinical reviews held with the patient present either by an individual provider or a multidisciplinary team [6].

Medication review services have been shown to positively impact patient outcomes, including economic, clinical, and ethical aspects. Economically, they can lead to significant cost savings for both patients and healthcare facilities by reducing hospital admissions and preventing adverse drug reactions [7,8]. Clinically, they improve disease-specific outcomes including reduce hospital readmissions [9]. These services improve the quality of life for chronic disease patients and enhance prescribing safety, although their effect on mortality is minimal [10,11].

Medication Reconciliation (MedRec) is another pharmacist-led intervention that plays an important role in promoting medication safety, particularly during transitions of care. It involves verifying and documenting a complete and accurate medication list to identify discrepancies across care settings [12]. While MedRec and medication review share overlapping goals, they serve distinct purposes: reconciliation ensures medication accuracy, while review focuses on optimizing therapy. MedRec is often a prerequisite to effective medication review, helping to ensure that the information assessed is complete and correct. However, this study focused exclusively on structured medication review practices, as defined by the PCNE. A detailed exploration of MedRec, which involves different workflows and objectives, was outside the scope of this research.

Despite the significant benefits of medication review for patient care, the implementation of Medication Reviews varies significantly across different healthcare settings and countries. In countries such as the United Kingdom and Australia, medication review services are well integrated into pharmacy practices and are backed by national policies and reimbursement schemes. Several barriers can limit the implementation and effectiveness of Medication Reviews [13]. Challenges include pharmacists’ lack of confidence and communication skills, minimal awareness among healthcare professionals about the evolving pharmacy role [13], and heavy workloads, reducing the focus on clinical tasks [14]. A study in Jordan (2022) identified barriers such as physician resistance to interventions, patients’ reluctance to share information, inadequate pharmacist training, lack of resources and IT infrastructure, and inconsistent regulations or remuneration for medication review services [15]. Given the unique cultural, educational, and healthcare context of the UAE, it is important to investigate these factors within the local setting.

In the UAE, the integration of medication reviews into routine pharmacy practice is still developing. There is limited research on pharmacists’ engagement and the factors influencing their practice, despite the healthcare system’s rapid growth and focus on patient safety. Technological advancements, including electronic medical records (EMRs), telehealth, e-prescribing, and clinical decision support systems (CDSS), have significantly transformed medication reviews, in response to inconsistencies caused by the COVID-19 pandemic [16]. Artificial intelligence (AI) is being increasingly utilized in medication order reviews [16]. Tools such as the Beers Criteria and STOPP/START aid in the review process for complex conditions. These innovations signify a shift toward more standardized and technology-driven practices.

Despite the growing interest in medication management in the UAE, significant gaps remain in understanding medication review practices among pharmacy professionals. Previous studies, including those by Alzubaidi et al. (2018) and Kharaba et al. (2022), have highlighted barriers such as time constraints, lack of organizational support, and ambiguity regarding pharmacists’ roles [17,18]. While these studies underscore the need for expanded clinical services, their primary focus has been on community pharmacists, leaving unexplored the practices and challenges faced by clinical pharmacists. Thus, a comprehensive understanding of medication review practices across different pharmacy settings in the UAE remains incomplete.

To date, no comprehensive investigation has assessed pharmacists’ knowledge, attitudes, practices, and perceived barriers (KAPB) related specifically to medication reviews in the UAE. Additionally, no studies have systematically examined the influence of structural factors, including educational gaps, lack of standardized tools, and insufficient policy frameworks. The present study aims to address this critical gap by evaluating the prevalence and nature of medication review practices among licensed pharmacists in the UAE, as well as identifying structural and educational barriers limiting their effectiveness. By including pharmacists from hospital, community, and clinical sectors, this research seeks to provide insights that could inform comprehensive national strategies aimed at improving medication safety, enhancing pharmacist competencies, and establishing standardized medication review protocols tailored specifically to the UAE’s healthcare context.

## Methods

### Study design

This cross-sectional study utilized an online survey to gather data from pharmacy professionals, including community pharmacists, hospital pharmacists, and clinical pharmacists, in the United Arab Emirates. The study included licensed and practicing pharmacists who held at least a bachelor’s degree in pharmacy and worked in community or clinical settings, provided that they were willing to provide informed consent. Pharmacy technicians, assistants, students, and non-practicing pharmacists were excluded from the study.

### Sample size determination

The following formula for the sample size was applied [19]:


n=N×X÷(X+N−1),

Where $X=Z_{{\alpha /\text{2}}^{\text{2}}}-*p*(\text{1}-p)/{MOE}^{\text{2}}$

and $Z_{\alpha /\text{2}}$ is the critical value of the normal distribution at α/2 (e.g., for a confidence level of 95%, α is 0.05 and the critical value is 1.96), *MOE* is the margin of error, p is the sample proportion, and *N* is the population size. Based on this formula and assuming a 50% unknown sample proportion, at least 385 respondents are needed for this study.

### Data collection

The survey instrument used in this study was specifically developed by the research team following a comprehensive review of the existing literature on medication review practices [13–16,20–30]. Key sources included studies by Al-Tameemi & Sarriff, Rendrayani et al., Abu Assab et al.,Bulajeva et al., De Oliveira Santos Silva et al., Graabæk & Kjeldsen, Harris & Argáez, Hussain & Babar, International Pharmaceutical Federation [FIP], Kilonzi et al., Pharmaceutical Care Network Europe, Ranchon et al.,Santos Júnior et al., Thiruchelvam et al., Wong & Sze, Yoo et al. The questionnaire was further refined through consultation with practicing pharmacists in the UAE to ensure contextual relevance.

The questionnaire was organized into five sections. The demographic section gathered information about participants’ occupations, years of experience, the emirates they work in, education levels, and certifications. The knowledge section assessed their familiarity with medication review concepts and awareness of their different types. Attitudinal questions were included to gauge their views and opinions on medication review. The barriers section investigated the obstacles faced and participants’ confidence in strategies to reduce medication errors. The medication review practices section examined the frequency, types, and conditions under which medication reviews were conducted. It also explored the number of medications reviewed, time spent, information considered, resources utilized, and the interventions identified and implemented. Additionally, the questionnaire included questions about common DRPs encountered, the perceived impact of medication review on patient outcomes, and overall satisfaction with MRP. This diverse content was designed to provide a comprehensive view of pharmacists’ experiences and practices related to medication review.

### Data collection procedures

The survey questionnaire was designed using Google Forms and was distributed between 25 March 2024 and 25 July 2024. It was shared via emails, LinkedIn, WhatsApp, Facebook groups, and other social media platforms, targeting pharmacists in the UAE. Additionally, researchers visited pharmacies and hospitals to distribute the survey link to potential respondents.

A combination of convenience and snowball sampling was employed. Convenience sampling was used due to its easily accessible distribution channels, while snowball sampling was applied as pharmacists were encouraged to share the questionnaire with their colleagues. Participants could only access the questionnaire after providing informed consent, which was obtained through a mandatory consent form on the first page. The consent form outlined the study’s purpose, benefits, and potential risks.

### Scoring and standardization of Knowledge, Attitude, and Practice (KAP)

The Knowledge, Attitude, and Practice (KAP) domains were scored separately and then standardized to facilitate direct comparison across domains. The Knowledge domain comprised nine true/false questions, each coded as correct (1) or incorrect (0), yielding a total score from 0 to 9. The Attitude domain included eight items rated on a 5-point Likert scale (1 = strongly disagree to 5 = strongly agree), with total scores ranging from 8 to 40.

The Practice domain consisted of many descriptive questions and nine quantified items into the practice component with varied response formats:

- Four binary Yes/No items with the first serving as a qualifying item. Participants needed to answer “Yes” to “Have you conducted any medication review during your practice?” to proceed;
- Four items rated on a 4-point frequency scale;
- One item asking how many DRPs were identified, with a range from 0 to 15.

Due to the heterogeneous response formats, each item was first individually rescaled to a 0–100% range based on its own minimum and maximum possible scores. For example, binary items were converted as 0 = 0% and 1 = 100%, while 4-point items were converted using:


$$POMP\;(Percentage\;of\;Maximum\;Possible)=\frac{Observed\;score-Minimum\;possible\;score}{Maximum\;possible\;score-Minimum\;possible\;score}\times \text{1 0 0}$$

The final Practice score was calculated as the mean of the nine rescaled item percentages, resulting in a total score ranging from 0% to 100%. This item-level percentage approach enabled harmonization across items with different formats and ensured that all domains (knowledge, attitude, and practice) were expressed on the same 0–100% scale.

### Data analysis

Statistical Package for the Social Sciences (SPSS) version 29 was used for data analysis. To assess face validity, the questionnaire was pilot tested on 5% of the sample size (n = 20). The pilot sample represented a diverse cross-section of pharmacy professionals working in community, hospital, and clinical settings across multiple emirates. This diversity reflects the broader target population of the study and supports the generalizability of the instrument.

Internal consistency was assessed using Cronbach’s alpha, with a threshold of 0.70 deemed acceptable. To improve internal consistency, one knowledge question was removed, and the scales of two practice questions were adjusted. After these changes, the Cronbach’s alpha reliability was found to be 0.749.

Construct validity for the KAP domains was assessed using exploratory factor analysis (EFA) with principal component extraction and Varimax rotation. Sampling adequacy was confirmed using the Kaiser–Meyer–Olkin (KMO) measure, and Bartlett’s test of sphericity verified suitability for factor analysis. Factor loadings of ≥0.40 were considered meaningful for interpretation; however, all items were retained in the analysis, as low-loading items that demonstrated acceptable internal consistency and significant correlations with their respective domain totals were considered conceptually relevant.

Exploratory factor analysis was conducted separately for each KAP domain. For the Knowledge domain, KMO was 0.730 and Bartlett’s test was significant (p < 0.001). Two factors explained 60.1% of the variance, and the total knowledge score correlated well with the factor scores (r = 0.631 and 0.746, p < 0.001). For the Attitude domain, KMO was 0.871 and Bartlett’s test was significant (p < 0.001). Two factors explained 59.5% of the variance, with strong correlations to the total attitude score (r = 0.811 and 0.581, p < 0.001). For the Practice domain, KMO was 0.673 and Bartlett’s test was significant (p < 0.001). A single factor explained 48.5% of the variance, and the total practice score was significantly correlated with the factor score (r = 0.669, p < 0.001). These results confirm sampling adequacy and factorability for all domains, and the factor solutions explained a substantial portion of variance. The significant correlations between domain scores and factor scores support the construct validity of the instrument.

The Kolmogorov–Smirnov (KS) test was employed to examine the data for normality, revealing that the data were not normally distributed. Frequencies and percentages were calculated for respondents’ sociodemographic characteristics, as well as their knowledge, attitudes, practices, and perceived barriers regarding medication review. To examine associations between sociodemographic factors and total knowledge, attitude, and practice scores, the Mann–Whitney U test and the Kruskal–Wallis H test were used for two-group and multi-group comparisons, respectively. For post hoc analyses following significant Kruskal–Wallis results, the Bonferroni correction was applied to adjust the significance level for multiple comparisons.

Effect sizes were calculated using rank-biserial correlation (r₍rb₎) for the Mann–Whitney U test and eta squared (η²) for the Kruskal–Wallis H test to estimate the magnitude of observed differences. Effect sizes were interpreted using conventional thresholds (e.g., 0.1 = small, 0.3 = moderate, 0.5 = large for r₍rb₎; 0.01 = small, 0.06 = moderate, 0.14 = large for η²). A p-value ≤ 0.05 was considered statistically significant unless adjusted by Bonferroni correction. Spearman’s rank-order correlation (ρ) was used to evaluate associations among knowledge, attitude, and practice scores, as these variables were continuous and non-normally distributed.

### Ethical considerations

Ethical approval was obtained from the Dubai Pharmacy College for Girls (DPCG) research and ethics committee (REC/FR/2023–24/07). Informed consent was obtained in written form via an online consent page at the beginning of the survey. This page clearly outlined the study’s objectives, procedures, voluntary participation, and confidentiality assurances, enabling participants to make an informed decision before proceeding. Participants provided explicit agreement by selecting a consent checkbox before accessing the survey. No personal identifiers were collected, and all the data was reported in aggregate form. Throughout the study, the researchers ensured confidentiality and followed ethical guidelines.

## Results

**Fig 1:**
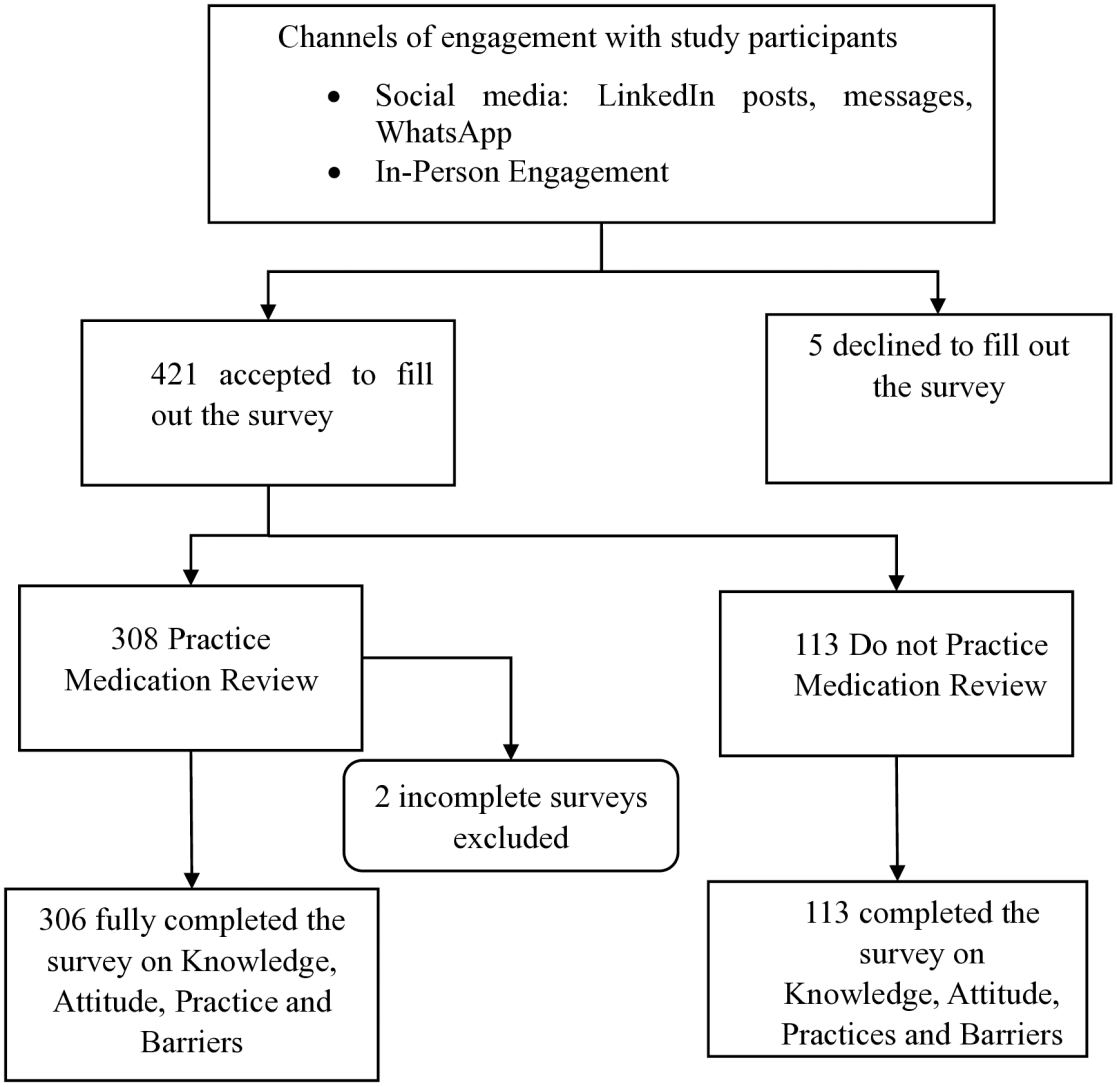
Workflow diagram illustrating the survey completion process by pharmacy professionals.

A total of 421 pharmacy professionals in the UAE responded to the survey, and all participants completed the knowledge, attitudes, and barrier components of the medication review survey. However, only 308 participants (73.2%) were eligible to complete the practice component, as they had conducted medication reviews during their practice. Of these 308 participants, only 306 fully completed the practice component, with 2 participants’ responses being incomplete and subsequently excluded from the analysis (Fig 1).

### Sociodemographic factors

More than half of the respondents were female (57.7%, n = 243). Most of the participants were aged 31–40 years (45.4%, n = 191). A significant majority were identified as Asian (72.0%, n = 303). Many of the respondents worked in retail/community pharmacies (35.4%, n = 149) and government hospitals (16.4%, n = 69), with nearly 40% having 1–5 years of experience.

Nearly half of the participants had a bachelor’s degree in pharmacy, with many holding advanced degrees: PharmD (24.0%, n = 101) and Post Graduate degree (27.6%, n = 116). Additionally, 16.6% (n = 70) were board certified (Table 1).

**Table 1:** Sociodemographic characteristics.

Sociodemographic Characteristics	Number (n = 421)	Percentage (%)
**Gender**		
Male	178	42.3
Female	243	57.7
**Age Groups (in years)**		
21–30	182	43.2
31–40	191	45.4
41–50	39	9.3
Above 51	9	2.1
**Nationality**		
Emirati	33	7.8
African^†^	78	18.5
Asian^‡^	303	72.0
Other^§^	7	1.7
**Role at Practice Setting**		
Clinical Pharmacist	78	18.5
Community Pharmacist	148	35.2
Inpatient Pharmacist	60	14.3
Outpatient Pharmacist	130	30.9
Pharmacist in Insurance Sector	5	1.2
**Type of Setting**		
**Hospitals**		
Government Hospitals	69	16.4
Semi Government Hospital	28	6.7
Private Non-Teaching Hospital	61	14.5
Private Teaching Hospital	50	11.9
**Clinics**		
Government Clinics	5	1.2
Private Clinics	57	13.5
Insurance Company	2	0.5
Retail/Community Pharmacy	149	35.4
**Years of Experience in Pharmacy**		
Less than 1 year	27	6.4
1 - 5 years	168	39.9
6 - 10 years	116	27.6
More than 10 years	110	26.1
**In Which Emirate Do You Currently Work**		
Abu Dhabi/Al Ain	114	27.1
Ajman	60	14.3
Dubai	115	27.3
Fujairah	19	4.5
Ras Al Khaimah	23	5.5
Sharjah	79	18.8
Umm Al Quwain	11	2.6
**What Is Your Highest Level of Education in Pharmacy**		
Bachelor’s degree in pharmacy	204	48.5
Doctor of Pharmacy (PharmD)	101	24.0
Post Graduate Degree	116	27.6
**Board Certification** ^**#**^	70	16.6

† Includes Algeria, Benin, Comoros, Egypt, Eritrea, Libya, Sudan, and Tanzania.

‡ Includes India, Iran, Iraq, Jordan, South Korea, Lebanon, Pakistan, Palestine, Syria, Yemen, and the Philippines.

§ Includes Armenia, Germany, Italy, Canada, Dominica, and Saint Kitts and Nevis.

# Footnote: Board certifications include Board Certified Oncology Pharmacist (BCOP), Board Certified Psychiatric Pharmacist (BCPP), Board Certified Pharmacotherapy Specialist (BCPS), Board Certified Nutrition Support Pharmacist (BCNSP), Board Certified Pain Management Pharmacist (BCPMP), Board Certified Pediatric Pharmacist (BCPPS), Board Certified Infectious Diseases Pharmacist (BCIDP), Board Certified Nuclear Pharmacist (BCNP), Board Certified Compounded Sterile Preparations Pharmacist (BCSCP), Board Certified Critical Care Pharmacist (BCCCP), Board Certified Solid Organ Transplant Pharmacist (BCSOT), Board Certified Ambulatory Care Pharmacist (BCACP), and Board Certified Cardiology Pharmacist (BCCP).

### Knowledge regarding medication review

**Fig 2:**
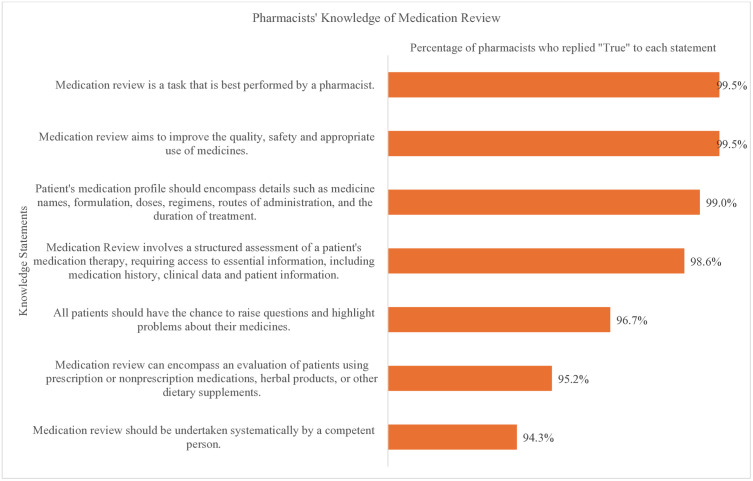
Pharmacists knowledge of medication review.

Fig 2 illustrates pharmacists’ knowledge of medication review based on agreement with key statements. The highest agreement (99.5%) was observed for the statements that medication review is best performed by a pharmacist and that it improves quality, safety, and appropriate use of medicines. The lowest agreement (94.3%) was reported for the need to conduct medication reviews systematically by a competent person. Overall, participants demonstrated a high level of knowledge across all domains.

**Fig 3:**
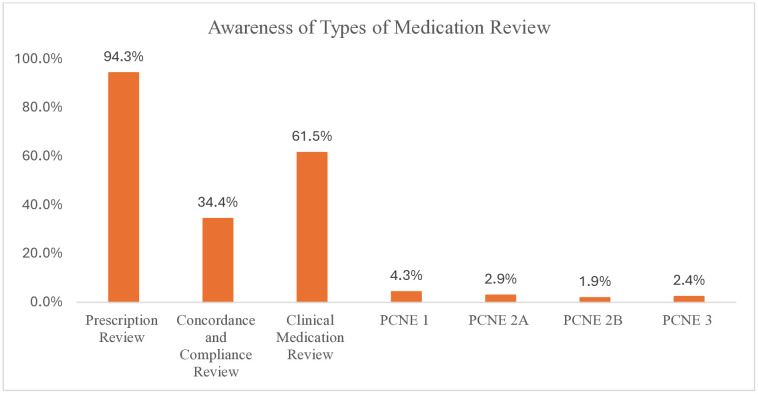
Awareness of medication review among pharmacy professionals.

Pharmacists demonstrated the highest awareness of prescription reviews at 94.3%, followed by clinical medication reviews at 61.5%, and concordance/compliance reviews at 34.4%. In contrast, awareness of PCNE classifications was significantly lower, with only 4.3% recognizing PCNE 1. Even fewer were aware of PCNE 2A (2.9%), PCNE 2B (1.9%), and PCNE 3 (2.4%). (Fig 3)

### Attitudes toward medication review

Table 2 shows the attitudes of the participants toward the Medication Review. The findings indicate a strong consensus on the benefits of medication reviews: 98.8% of respondents agree that these reviews improve patient outcomes, 96.9% believe that they enhance satisfaction and adherence, and 96.2% recognize the advantages of interprofessional collaboration. Despite time constraints, most pharmacists find this practice valuable (95.1%) and feel competent in conducting reviews (95.3%). Furthermore, many expressed a desire for additional training (91.5%). Financial incentives are viewed as a motivating factor by 85.8% of the participants.

**Table 2:** Pharmacists’ attitudes toward medication review.

Statements	Agree (n, %)	Neutral (n, %)	Disagree (n, %)
Involving patients in the medication review process positively impacts its outcomes.	398 (94.6%)	20 (4.8%)	3 (0.7%)
Medication reviews significantly improve patient-specific outcomes, such as disease management and overall health.	416 (98.8%)	5 (1.2%)	0 (0.0%)
Interprofessional collaboration contributes to the success of medication review practices.	405 (96.2%)	15 (3.6%)	1 (0.2%)
Despite time constraints, medication review practices are a valuable investment in patient care.	400 (95.1%)	20 (4.8%)	1 (0.2%)
Medication review practices can lead to improved patient satisfaction or adherence.	408 (96.9%)	13 (3.1%)	0 (0.0%)
I believe I can effectively conduct medication reviews as part of my professional responsibilities.	401 (95.3%)	18 (4.3%)	2 (0.4%)
Financial incentives or reimbursement can increase my willingness to perform medication review services.	361 (85.8%)	40 (9.5%)	20 (4.8%)
I would like to be trained in the medication review process.	385 (91.5%)	33 (7.8%)	3 (0.7%)

### Perceived barriers to medication review

**Fig 4:**
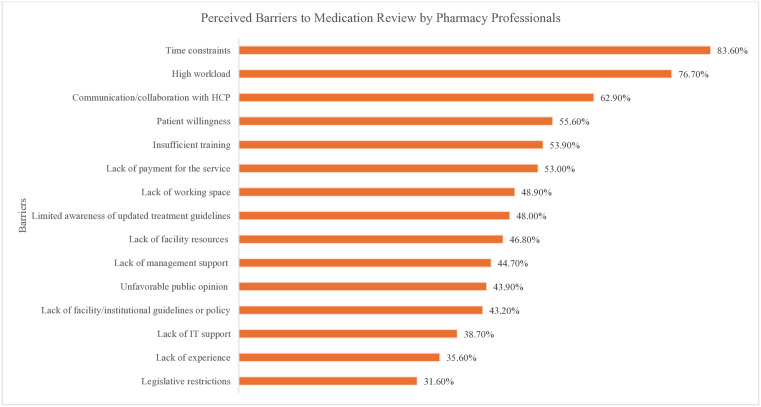
Perceived Barriers. **Abbreviations:** HCP – health care professionals.

The most cited barrier to conducting medication reviews was time constraints, reported by 83.6% of the pharmacists. This was closely followed by a high workload, noted by 76.7% of the respondents. Other significant barriers included challenges in communication and collaboration with healthcare professionals (62.9%), inadequate training (53.9%), patient willingness (55.6%), and lack of payment for the service (53.0%) (Fig 4).

### Practices related to medication review

#### Types of reviews.

Approximately 73% of pharmacy professionals reported conducting medication reviews. The most performed type was prescription reviews with complete medical records (62.7%). Clinical medication reviews, which involve both the presence of the patient and access to medical notes, were conducted by 42.2% of pharmacists. Compliance reviews with complete records were performed by 40.8%, while concordance reviews, focusing on patient–provider agreement with full documentation, were the least common, reported by only 22.2% of participants. Additionally, 44.8% of pharmacists conducted prescription reviews without complete medical records, and 35.9% engaged in unplanned, unstructured reviews without the patient present.

#### Information and resources considered for medication review.

When asked about the information and resources considered for medication reviews, 91.5% of the pharmacists indicated that medication history was the most important factor. Patient information and consultations with general practitioners (GPs) were also highly valued, each receiving 88.2% of the responses. Additionally, 78.8% of the pharmacists reported utilizing clinical data in their review process. However, only approximately half of the respondents found other resources necessary: 59.2% reported using tertiary resources, 39.5% used secondary sources, and 37.9% relied on primary resources.

#### Strategies used in conducting medication reviews.

**Fig 5:**
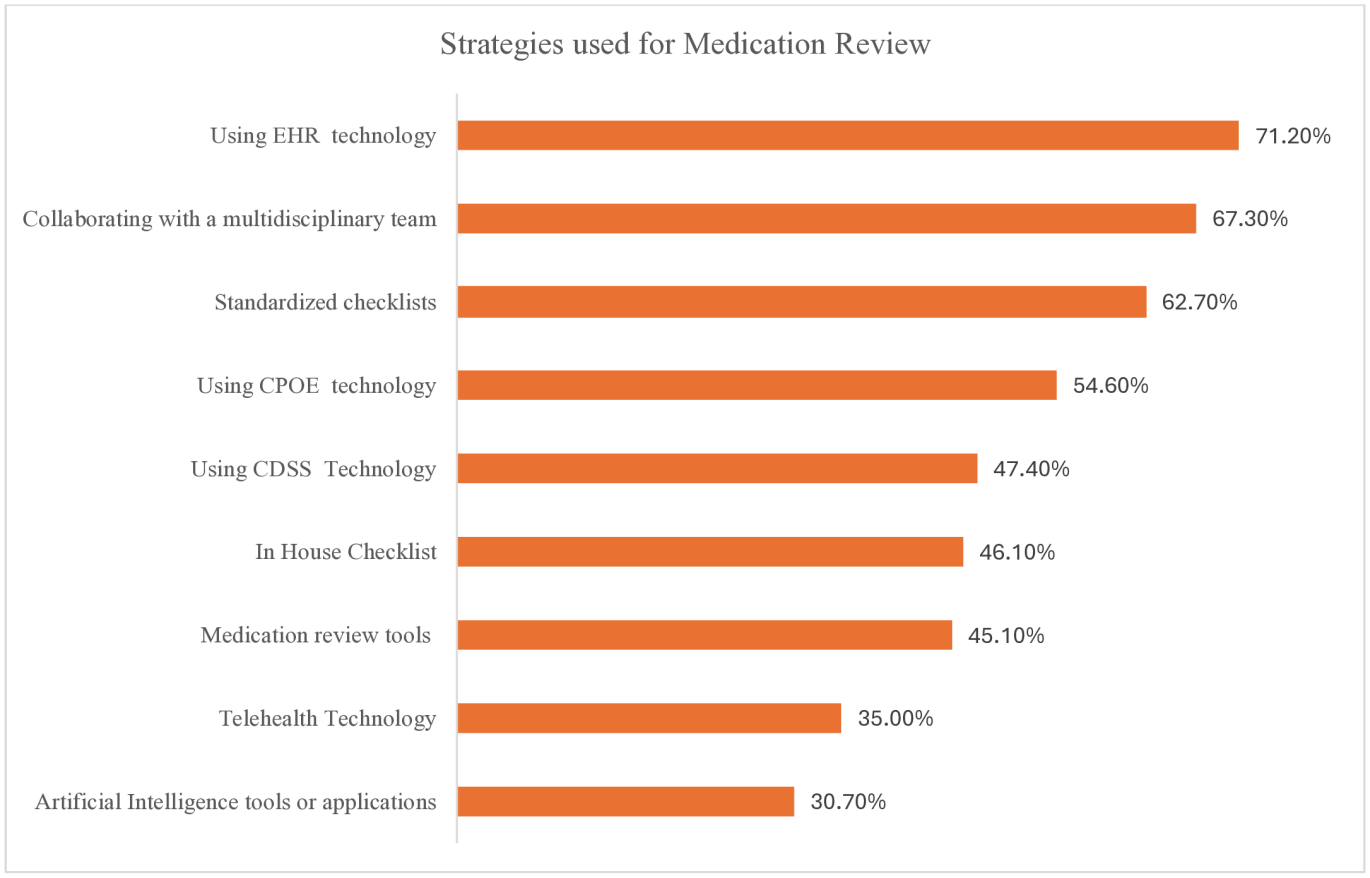
Strategies used in conducting medication reviews. **Abbreviations**: EHR – Electronic Health Record, CPOE – Computerized Physician Order Entry, CDSS – Computerized Decision Support System.

As shown in Fig 5, electronic medical record or electronic health record (EMR/EHR) documentation tools were used by 71.2% of the pharmacists. Additionally, 67.3% of the surveyed respondents also engage in interprofessional collaboration. Among the checklist and review tools, standardized checklists were used by 62.7% of the pharmacists, followed by in-house (46.1%) and medication review tools (45.1%), respectively. Technology-driven strategies, such as CPOE (Computerized Physician Order Entry) and CDSS (Computerized Decision Support System) were utilized by 54.6% and 47.4% of respondents, respectively. On the other hand, telehealth and AI (Artificial Intelligence) were the least commonly used, reported by 35.0% and 30.7% of participants, respectively.

**Fig 6:**
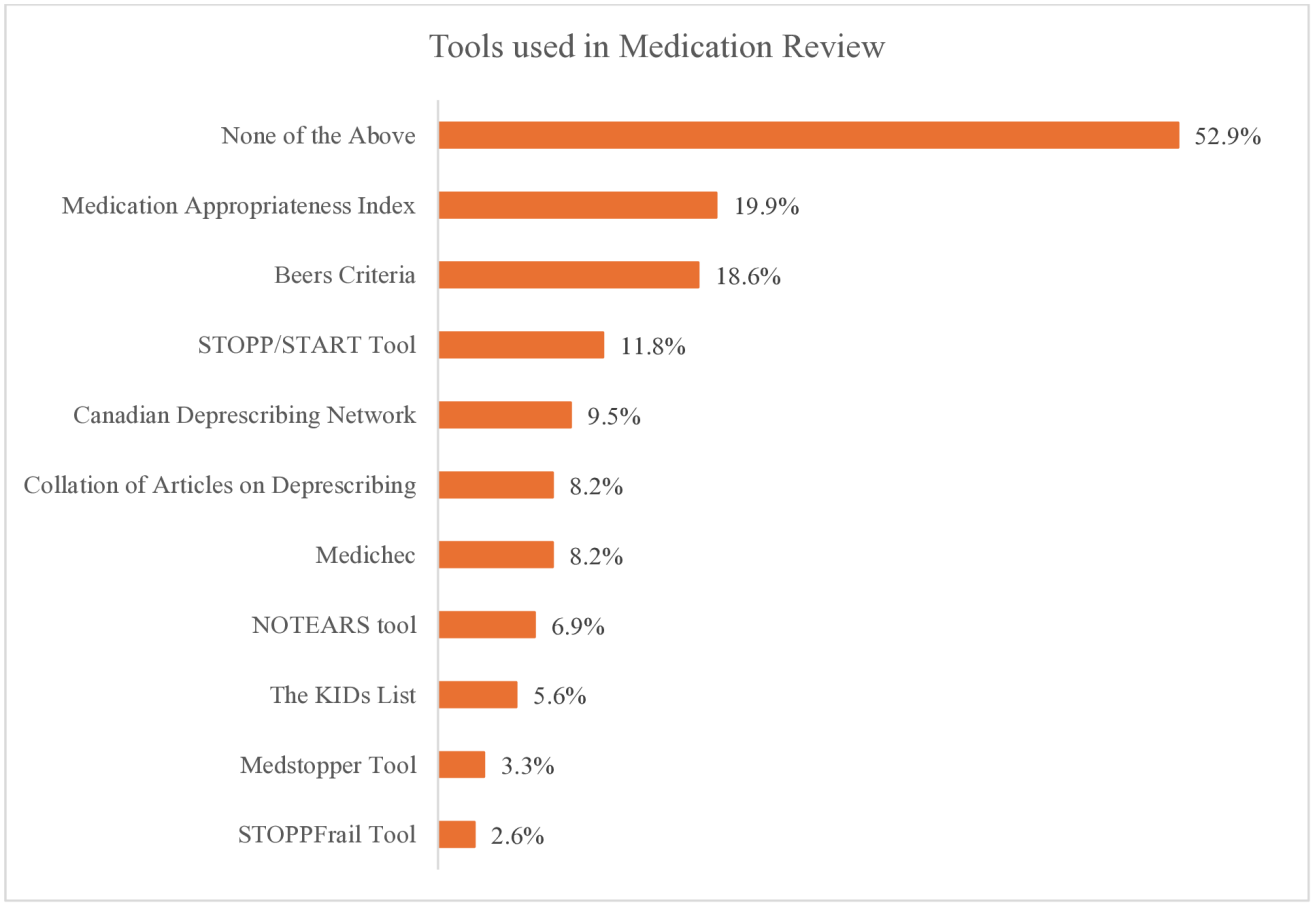
Review Tools used in Conducting Medication review. **Abbreviations:** KIDs - Key Potentially Inappropriate Drugs in Pediatrics; NOTEARS – Need and indication, Open questions, Tests and monitoring, Evidence and guidelines, Adverse events, Risk reduction or prevention, Simplification and switches; START – Screening Tool to Alert to Right Treatment; STOPP – Screening Tool Of Older People’s Prescriptions; STOPPFRAIL – Screening Tool of Older Persons Prescriptions in Frail adults with limited life expectancy.

Fig 6 shows the use of medication review tools used by the respondents. More than half of the respondents (55.9%) did not use any of the tools. A few of the participants used tools. The most common tools used are the medication appropriateness index (19.9%), beers criteria (18.6%) and the STOPP/START tool (11.80%).

#### Types of patients or conditions typically addressed.

Among the pharmacy professionals surveyed, 47.1% reported conducting medication reviews for all patients, emphasizing the importance of universal medication oversight. Reviews were most frequently conducted for patients with potentially serious or common side effects (45.1%) and those who were frail or had multiple illnesses (41.5%). Additionally, 35% of the respondents provided reviews for pregnant or breastfeeding patients (Table 3).

**Table 3:** Types of Patients or Conditions Typically Addressed.

Type of Patient or Condition	Frequency (Yes)	Percentage
Frail or Have Multiple Illnesses	127	58.5%
Potentially Serious or Common Side Effects	138	45.1%
Prescribed Controlled Substances	90	29.4%
Requires Regular Monitoring	59	19.3%
Polypharmacy	104	34%
Geriatric Patients	103	33.7%
Pediatric Patients	103	33.7%
Pregnant and/or Breastfeeding Patients	107	35%
Allergies	106	34.6%
Genetic Factors	46	15%
Drug Interactions	96	31.4%
All Patients	92	30%

#### Average time required per patient for medication review.

**Fig 7:**
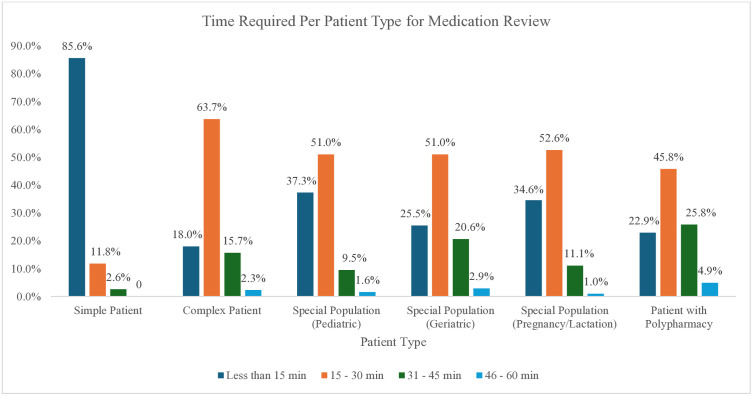
Average time to conduct Medication Review.

The majority of the pharmacists reported that simple patients required the least amount of time for medication reviews, with 85.6% of the pharmacists typically spending less than 15 minutes. In contrast, pharmacists reported that medication reviews for certain patient groups typically required more than 15 minutes. These groups included critically ill patients, those with polypharmacy, and special populations such as pediatrics, geriatrics, pregnant patients, and lactating patients. (Fig 7)

#### Issues identified during medication review.

**Fig 8:**
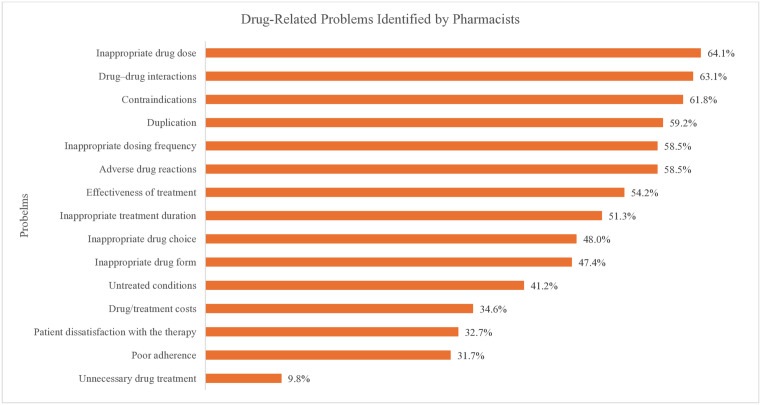
Drug-related problems identified by pharmacy professionals.

Among the surveyed pharmacy professionals, 61.8% reported having identified contraindicated drugs prescribed at some point in their practice, whereas 58.5% encountered adverse drug reactions. Drug–drug interactions (63.1%) and inappropriate drug dosing (64.1%) were also frequently identified issues. Additionally, 59.2% noted instances of drug duplication, and 54.2% reported challenges in treatment effectiveness. Reviews of medication also included identifying inappropriate treatment duration (51.3%), untreated conditions (41.2%), poor adherence (31.7%), and patient dissatisfaction with therapy (32.7%) (Fig 8).

Among the pharmacists surveyed, 99.3% reported acting on identified DRPs following medication reviews. Specifically, 84.3% contacted the prescribing physicians directly, 12.1% advised patients to consult their physicians, and 2.9% offered alternatives to patients. Among the respondents, 65% of the pharmacists conducted regular follow-ups on patients’ drug therapy, 73.9% developed strategies to prevent or resolve DRPs, and 74.2% documented their interventions. The institutional outlook on the stronger implementation of drug therapy practices is generally positive (73.2%), with 15.4% expressing a neutral attitude, 5.9% negative, and 5.6% unsure.

### Differences in knowledge, attitudes, and practices across sociodemographic characteristics

Differences in knowledge, attitudes, and practices across sociodemographic characteristics are presented in Table 4. Knowledge and attitude differed significantly by gender (*p* = 0.003 and 0.007, respectively), whereas practice did not (*p* = 0.598). No significant differences were detected across age groups or nationalities in any domain. Practice scores, however, varied by pharmacists’ professional role and practice setting (both *p* < 0.001) and were higher among pharmacists with more years of experience (*p* = 0.001), those holding board certification (*p* = 0.018), those working in particular emirates (*p* = 0.021), and those with more advanced pharmacy degrees (*p* = 0.027). Post-hoc tests indicated that clinical pharmacists outperformed community (adjusted p < 0.001) and outpatient pharmacists (adjusted p = 0.003). Hospital-based pharmacists showed higher knowledge and practice scores than those in retail/community settings (adjusted p = 0.001 and < 0.001, respectively). Pharmacists with > 10 years of experience scored higher in practice than those with 6–10 years (adjusted p = 0.001) and 1–5 years (adjusted p = 0.014).

**Table 4:** Differences in knowledge, attitudes, and practices across sociodemographic characteristics.

Sociodemographic Characteristics	Knowledge				Attitude				Practice			
	Median (IQR)	Z^a^	X^2^ (df)^b^	p value	Median (IQR)	Z^a^	X^2^ (df)^b^	p value	Median (IQR)	Z^a^	X^2^ (df)^b^	p value
**Gender**												
Male	77.7(0)	3.014		0.003*	90.6(18.7)	2.685		0.007*	65.5(18.3)	0.528		0.598
Female	77.7(0)				93.7(12.5)				67.2(24.2)			
**Age Groups (in years)**												
21–30	77.7(0)		1.142 (3)	0.767	93.7(17.2)		0.229 (3)	0.973	63.3(20.9)		7.806(3)	0.050
31–40	77.7(0)				93.7(15.6)				68.6(207)			
41–50	77.7(0)				90.6(15.6)				67.7(23.3)			
Above 51	77.7(0)				92.2(14.8)				75.8(11.6)			
**Nationality**												
Emirati	77.7(0)		4.479(3)	0.214	93.7(12.5)		1.152 (3)	0.765	68.8(24.4)		3 (3)	0.582
African^†^	77.7(0)				93.7(12.5)				67.7(23.3)			
Asian^‡^	77.7(0)				93.7(15.6)				66.1(20.8)			
Other^§^	77.7(13.5)				98.4(16.4)				71.1(42.6)			
**Role at Practice Setting**												
Clinical Pharmacist	77.7(0)		7.587 (4)	0.108	96.8(9.4)		6.185(4)	0.186	73.3(28.8)		24.998(4)	<.000**
Community Pharmacist	77.7(0)				90.6(18.7)				62.7(20.5)			
Inpatient Pharmacist	77.7(0)				93.7(15.6)				67.2(22.2)			
Outpatient Pharmacist	77.7(0)				93.7(15.6)				63.2(20.1)			
Pharmacist in Insurance Sector	77.7(.)				84.4(.)				61.6(.)			
**Type of Setting**												
Hospitals	77.7(11.1)		26.299 (3)	<0.001**	90.6(21.1)		9.798 (3)	0.020*	69.5(22.2)		27.315(3)	<0.000**
Clinics	77.7(0)				93.7(12.5)				63.3(24.4)			
Insurance Company (1)												
Retail/Community Pharmacy	77.7(0)				90.6(18.7)				58.8(20.5)			
**Years of Experience in Pharmacy**												
Less than 1 year	77.7(0)		1.337 (3)	0.720	96.9(25)		3.446 (3)	0.328	58.8(24.4)		17.147(3)	0.001**
1 - 5 years	77.7 (0)				93.7(18.7)				65.5(20)			
6 - 10 years	77.7 (0)				96.9(12.5)				63.8(19.3)			
More than 10 years	77.7 (0)				93.7(15.6)				72.2(19.8)			
**In Which Emirate Do You Currently Work**												
Abu Dhabi/Al Ain	77.7(0)		13.325 (6)	0.038*	93.7(14.8)		8.183 (6)	0.225	69.7(22.6)		14.855(6)	0.021*
Ajman	77.7(0)				93.7(12.5)				65()			
Dubai	77.7(0)				93.7(15.6)				66.3(23.6)			
Fujairah	77.7(0)				87.5(17.2)				63.8(31.8)			
Ras Al Khaimah	77.7(0)				96.9(9.4)				73.3(11.1)			
Sharjah	77.7(0)				90.6(24.2)				59.7(18.7)			
Umm Al Quwain	77.7(11.1)				90.6(23.4)				63.8(30.8)			
**What Is Your Highest Level of Education in Pharmacy**												
Bachelor’s Degree in Pharmacy	77.7(0)		2.36 (2)	0.308	90.2(15)		2.36 (2)	0.308	63.6(21.4)		7.248(2)	0.027*
Doctor of Pharmacy (PharmD)	77.7(0)				93.7(18)				67.5(16.5)			
Post Graduate	77.7(0)				93.7(12)				70(23.1)			
**Board Certification Status**												
**Board Certification** ^**#**^	77.7(0)	0.862		0.389	96.8(9.4)	1.663		0.096	70(28.8)	2.360		0.018*
**No Board certification**	77.7(0)				93.7(15.6)				66.1(20.5)			

**Note: a** Mann‒Whitney U test; **b** Kruskal‒Wallis H test.

^†^Includes Algeria, Benin, Comoros, Egypt, Eritrea, Libya, Sudan, and Tanzania.

^‡^Includes India, Iran, Iraq, Jordan, South Korea, Lebanon, Pakistan, Palestine, Syria, Yemen, and the Philippines.

^§^Includes Armenia, Germany, Italy, Canada, Dominica, and Saint Kitts and Nevis.

*p-value significant (p-value <0.05).

** Statistically significant (Bonferroni correction applied for multiple comparisons).

(1)Only one respondent from this group; data not included in statistical comparison.

# Footnote: Board certifications include Board Certified Oncology Pharmacist (BCOP), Board Certified Psychiatric Pharmacist (BCPP), Board Certified Pharmacotherapy Specialist (BCPS), Board Certified Nutrition Support Pharmacist (BCNSP), Board Certified Pain Management Pharmacist (BCPMP), Board Certified Pediatric Pharmacist (BCPPS), Board Certified Infectious Diseases Pharmacist (BCIDP), Board Certified Nuclear Pharmacist (BCNP), Board Certified Compounded Sterile Preparations Pharmacist (BCSCP), Board Certified Critical Care Pharmacist (BCCCP), Board Certified Solid Organ Transplant Pharmacist (BCSOT), Board Certified Ambulatory Care Pharmacist (BCACP), and Board Certified Cardiology Pharmacist (BCCP).

Type of practice setting (η² = 0.081), professional role (η² = 0.073), and years of experience (η² = 0.047) each showed small-to-medium associations with practice. Practice setting also displayed a medium association with knowledge (η² = 0.077) and a small association with attitude (η² = 0.023). Board certification correlated positively with attitude and practice, while gender produced only small differences in knowledge and attitude. Age group, nationality, and education level were not significantly related to any domain. Full statistical details and effect size comparisons are provided in Supplementary S2 Table.

### Correlations among knowledge, attitudes and practices regarding medication review

The correlations between knowledge, attitudes, and practices among participants were analyzed via Spearman’s rank-order correlations. A weak but significant positive association was noted between knowledge and attitude (r = 0.20, *p* = 0.001) and between attitude and practice (r = 0.16, *p* = 0.004), while the knowledge–practice association was small and not statistically significant (r = 0.10, *p* = 0.088).

## Discussion

This study assessed the knowledge, attitudes, and practices of pharmacists in the UAE regarding medication reviews. Our findings provide valuable insights into the current state of medication review practices in the country. The sociodemographic data of the participating pharmacists aligns with a previous study that reveals most of the pharmacists in the UAE are females and are less than 45 years of age [31].

Our results show a strong consensus among pharmacists regarding the importance of medication reviews being pharmacist-led. The participants emphasized the need for comprehensive patient profiles and structured assessments that include complete clinical data. Almost all the participants recognized the pharmacist’s critical role in enhancing patient care through medication reviews aligning with international standards that advocate pharmacist-led medication reviews to enhance medication safety and efficacy [26,32]. These findings are consistent with earlier studies that have documented similar perspectives [28,21].

Awareness of the different types of medication review is limited among pharmacists in our survey. A study by Wong and Sze et al. (2021) revealed that 38.6% of pharmacists were unaware or unsure of the prescription review, compliance and concordance review, and clinical medication review [28,21]. Particularly, our study participants had limited knowledge regarding the Pharmaceutical Care Network Europe (PCNE) types of medication review. Only 4.3% of participants recognized PCNE 1, and even fewer were familiar with the PCNE 2A, 2B and 3 classifications. The absence of a widely adopted classification system represents a major barrier to standardizing and documenting the quality of clinical pharmacy services, making objective evaluation and benchmarking of medication review services difficult within the UAE healthcare system. Awareness of pharmacists regarding the PCNE classification system has not been widely reported previously, making this study a valuable contribution [33].

The present study reveals a positive attitude among pharmacists toward medication review. Most of the respondents agreed that medication reviews improve patient outcomes, enhance patient satisfaction and adherence, and foster interprofessional collaboration. This is consistent with studies in other countries where pharmacists recognized the benefits of medication reviews despite potential barriers. A cross-sectional study by Alshehri et al. [34], along with studies conducted in Jordan and Lebanon, reported similar positive attitudes among pharmacists [35,36]. The participants acknowledged the critical role of interprofessional collaboration in ensuring the effectiveness of medication reviews. In support of this, a study conducted in Germany demonstrated that implementing an interprofessional medication management program significantly enhanced patients’ sense of safety regarding their medication use [37].

Time constraints, high workloads, communication challenges with other healthcare professionals and inadequate training were cited as barriers. Several studies in the past have revealed barriers to the provision of medication review services in community and hospital settings [20,38–41]. Consistent with our study, insufficient time was the most frequently reported barrier to providing medication review services. Additionally, inadequate staffing, compensation, and training are commonly reported in various other studies, as highlighted in a systematic review [21]. Our findings reflect global challenges in the implementation of medication review services. As noted by Rose et al. [42], there is a lack of standardized definitions and frameworks internationally, which limits consistency in practice. European studies similarly report wide variations in service levels, pharmacist involvement, and remuneration. These insights highlight the need for clear policies, professional training, and system-level support to enhance knowledge, attitudes, and practices around medication review globally [22,43]. Addressing these barriers is crucial for the successful implementation of medication review by pharmacists.

Despite the proven benefits of pharmacist-led medication review in reducing DRPs, its routine integration into UAE healthcare practice remains limited like other parts of the world. Policy-level interventions form a cornerstone for enabling widespread adoption. Incorporating medication review requirements into national health policies and accreditation frameworks mandated by MOHAP/DOH/DHA like that of the Joint Commission International (JCI) can drive institutional accountability [44,45]. Although there are increasing numbers of hospital becoming JCI accredited where medication review is conducted, not all the hospitals are JCI accredited. Community and retail pharmacy accreditation does not have explicitly defined medication review criteria during accreditations. Further, aligning medication review with pharmacist performance indicators and reimbursement can incentivize active engagement, especially in both public and private hospital settings.

Equally important is capacity building through structured training programs. Targeted continuing professional development (CPD) sessions, covering PCNE classification, medication reconciliation skills, and effective interprofessional communication, are essential for empowering pharmacists [46]. The use of simulation-based learning, OSCE-styled training modules, and peer-led clinical case reviews can help build competence [47].

Addressing workflow challenges is key to embedding medication review within daily pharmacy practice. One effective approach is to digitize the process by integrating medication review documentation templates with drop-down PCNE coding directly into EHRs [48]. Workflow redesign, including allocating protected time slots and automating reviews at high-risk transition points (e.g., admission, discharge), ensures consistency and reduces time-related barriers.

Pilot projects and quality improvement (QI) initiatives can serve as a blueprint for wider implementation. Starting review services in high-yield areas such as intensive care units (ICU), geriatrics, or nephrology can provide measurable outcomes. Data from these pilots, when coded using standardized tools like PCNE, can be analyzed to evaluate impact and inform scalable models [49].

Our research indicates that most pharmacists (85.6%) spend less than 15 minutes on straightforward medication review, consistent with the findings of Merks et al. for follow-up visits (16.4 minutes), although the initial visits took longer times (21.5 minutes). For more complex cases, 63.7% of the pharmacists in our study spent between 15–30 minutes, however Merks et al. reported longer durations, with comprehensive reviews, averaging 40.8 minutes for first visits [50]. It has been well reported that complex cases, such as those involving polypharmacy and special populations, require more time [50,51]. Research by Duncan et al. reported that workload pressures can lead to quicker, often remote reviews without patient involvement, a factor not examined in our study. These variations highlight differences in practice thoroughness across studies.

In practice, about 73% of the pharmacists in our study reported conducting medication reviews, most commonly as prescription reviews, followed by clinical medication reviews or compliance reviews, and concordance reviews were the least common. This suggests that while pharmacists are engaging in some form of medication review, these activities are often limited in scope and may not reflect the comprehensive, patient-centered approach recommended by international standards. This also highlights the discrepancy between the high rate of self reported practice and the relatively low use of standardized tools, which likely reflects informal or unstructured review processes. Possible reasons include the absence of standardized national protocols, limited training, time constraints, and competing workload demands. Similar barriers have been reported in previous studies, such as the work by Chung et al., which showed that comprehensive medication management improves outcomes but is hindered by inconsistent protocols, scarce resources, lack of training, poor reimbursement, and low patient engagement [52].

EHR technology was the most common strategy used by pharmacy professionals in our study. Most healthcare facilities in the UAE have adopted EMR/EHR systems for patient care-related decision making, including medication review [53,54].However, the use of artificial intelligence and telehealth tools for conducting medication reviews remains limited. Only a few participants in our study utilized tools such as the Medication Appropriateness Index or the Beers Criteria. The limited use of these tools may affect the thoroughness and effectiveness of medication reviews. Encouraging the adoption of standardized tools could enhance the consistency and quality of medication review practice, as evidenced by improved outcomes in settings where such tools are routinely used [42].

Sociodemographic factors influence knowledge and attitudes toward medication reviews. In our study, females had greater knowledge and more positive attitudes toward medication review than males did. Our findings are consistent with similar studies that showed that women are more likely to possess greater knowledge regarding medication therapy management than men [20,28,21]. Possible reasons for better knowledge among females may include sociocultural influences, differing clinical exposures, and greater access to women-focused training, which could foster higher awareness and more positive attitudes. These differences warrant further qualitative exploration [55,56]. These differences highlight the need for targeted educational interventions to ensure adequate medication reviews.

In the present study, the practice setting significantly influenced pharmacists’ knowledge, attitudes and practices. Medication review was less common in community pharmacies compared to other practice settings in our study. This contrasts with Kharaba et al. (2022), who found that UAE community pharmacists spend more time interacting with patients than hospital pharmacists, emphasizing the greater importance and frequency of medication reviews [18]. In this study, factors such as practice setting, years of experience, emirate of practice, and education levels significantly impacted medication review practices. These findings do not completely align with those of a study conducted in Malaysia, which indicated that only the age of the pharmacist and years of experience were important in explaining differences in practices [28]. However, a survey conducted in Jordan reported results similar to those of our study, highlighting significant differences in medication review practices on the basis of education level and practice setting [57]. These variations in practice may be influenced by cultural factors.

In our study, a weak but statistically significant positive correlation was noted between attitudes and knowledge and between attitudes and practices. However, the correlation between knowledge and practices was negligible and not statistically significant. These findings indicate that while an improved attitude toward medication reviews is modestly associated with higher knowledge levels, such knowledge does not directly translate into better practice behaviors. Additionally, pharmacists with favorable attitudes are slightly more likely to demonstrate improved practices. Although statistically significant, the strength of association was weak and hence should be interpreted with caution regarding their practical impact on everyday practice Our results align in part with those reported by Wong et al. among Malaysian private hospital pharmacists regarding medication review, where a moderate positive correlation between knowledge and attitudes was observed (r = 0.467, p < 0.001). In their study, attitudes were positively associated with practice behaviors, but knowledge alone did not significantly predict practice performance. Wong et al. similarly observed no significant correlation between knowledge and practices, highlighting that knowledge alone might not drive meaningful behavioral changes [28]. Local studies in the UAE among pharmacists reported significant correlations between knowledge and attitudes related to other pharmacy-led services like pharmacovigilance and medication reconciliation [58,59]. While medication reconciliation is another important pharmacist-led service that supports medication safety, especially during transitions of care, it was not the focus of this study. Our investigation was limited to structured medication reviews, based on PCNE classifications, which differ in purpose and implementation. Nonetheless, the line between medication review and reconciliation may overlap in real-world practice, and future research could explore how these services are integrated within UAE pharmacy settings.

This study has several limitations. The self-reported nature of the survey may introduce response bias, with participants potentially overestimating their knowledge and practices. Although we tried to reach most of the pharmacists in the UAE, we might still have not reached the pharmacists who are not active on social media and in remote parts of the country. Additionally, the study was conducted in the UAE, and the findings may not be generalizable to other regions with different healthcare systems and pharmacist roles.

## Conclusion

The positive attitudes toward medication reviews among pharmacists have the potential to increase the adoption of comprehensive medication review practices in the UAE. However, barriers such as time constraints, high workloads, and the lack of standardized tools must be addressed. Implementing policies to allocate dedicated time for medication reviews, providing targeted educational interventions on medication review frameworks such as the PCNE classification system, and promoting consistent use of standardized tools could significantly enhance medication review practices. Interprofessional collaboration emerged as both a facilitator and a barrier; thus, establishing better communication channels between pharmacists and other healthcare professionals is essential. Interventions such as multidisciplinary team meetings and shared electronic health records could further support effective collaboration [60]. Financial incentives were viewed as motivating by 85.8% of the participants. Hence, introducing reimbursement models for medication review may encourage pharmacists to invest time and resources in conducting thorough reviews. Evidence from other healthcare systems suggests that compensation can increase the frequency and quality of medication reviews [61].

This study highlights the importance of medication reviews conducted by pharmacists in the UAE, demonstrating a strong recognition of their value and a positive attitude toward the implementation of medication reviews. However, our research also identified significant barriers, such as limited awareness of various types of medication reviews, inconsistent use of standardized tools, and insufficient familiarity with emerging technologies such as AI and telehealth. Gender differences in attitudes further underscore the need for a deeper exploration of pharmacists’ perceptions of medication reviews. To improve medication safety and patient outcomes, targeted educational interventions that integrate both knowledge enhancement and attitude-focused training are recommended. Standardizing the medication review process using frameworks like PCNE, coupled with promoting technological integration, can encourage consistent practices across pharmacy settings. Policymakers in the UAE play a critical role in ensuring that pharmacists receive adequate resources, dedicated training, and support to conduct safe, effective medication reviews, ultimately enhancing patient care and adherence outcomes.

## Supporting information

S1 Table STROBE checklist. (DOCX)

S2 Table Effect Size results for knowledge, attitude and practice. (DOCX)

S1 File Minimal data set. (XLSX)
