# Supplementary material for: Assessment of medication review among pharmacy professionals in the UAE: A cross-sectional study on knowledge, attitudes, practices, and barriers
Source: PLoS One. 2025 Nov 18;20(11):e0337233. doi: 10.1371/journal.pone.0337233 (PMC12626331; doi:10.1371/journal.pone.0337233)
Supplement: S2 Table — (DOCX) [file pone.0337233.s002.docx]

**Effect Size Results for Knowledge, Attitude and Practice**

**Rank-Biserial Correlation (r₍rb₎) and Mann–Whitney U Test Results for Knowledge, Attitude, and Practice Domains by Gender and Board Certification**

| **Independent Variable** | **Domain** | **U Statistic** | **p-value** | **r₍rb₎** | **Interpretation** |
| --- | --- | --- | --- | --- | --- |
| **Gender** | Knowledge | 12,765 | 0.003 | 0.110 | Small |
|  | Attitude | 13,529 | 0.007 | 0.177 | Small-to-moderate |
|  | Practice | 11,909.5 | 0.598 | 0.035 | Negligible |
| **Board Certification** | Knowledge | 7948.5 | 0.389 | 0.038 | Negligible |
|  | Attitude | 8677.0 | 0.096 | 0.134 | Small |
|  | Practice | 9131.5 | 0.018 | 0.193 | Small |

**Eta-Squared (η²) and Kruskal–Wallis H Test Results for Knowledge, Attitude, and Practice Domains by Demographic and Professional Characteristics**

| **Independent Variable** | **Domain** | **H (Test Stat)** | **df** | **p-value** | **η² (eta-squared)** | **Effect Size Interpretation** |
| --- | --- | --- | --- | --- | --- | --- |
| **Age Group** | Knowledge | 1.142 | 3 | .767 | ≈ 0.000 | No effect |
|  | Attitude | 0.229 | 3 | .973 | ≈ 0.000 | No effect |
|  | Practice | 7.806 | 3 | .050 | 0.016 | Small |
| **Nationality** | Knowledge | 4.479 | 3 | .214 | 0.005 | Negligible |
|  | Attitude | 1.152 | 3 | .765 | ≈ 0.000 | No effect |
|  | Practice | 1.953 | 3 | .582 | ≈ 0.000 | No effect |
| **Role at Practice Setting** | Knowledge | 7.587 | 4 | .108 | 0.012 | Small |
|  | Attitude | 6.185 | 4 | .186 | 0.007 | Negligible to small |
|  | Practice | 24.998 | 4 | 0.000 | 0.073 | Moderate |
| **Type of Practice Setting** | Knowledge | 26.299 | 3 | < .001 | 0.077 | Moderate |
|  | Attitude | 9.798 | 3 | .020 | 0.023 | Small |
|  | Practice | 27.315 | 3 | 0.00 | 0.081 | Moderate |
| **Years of Experience** | Knowledge | 1.337 | 3 | .720 | ≈ 0.000 | No effect |
|  | Attitude | 3.446 | 3 | .328 | 0.002 | Negligible |
|  | Practice | 17.147 | 3 | .001 | 0.047 | Small |
| **Emirate of Practice** | Knowledge | 13.325 | 6 | .038 | 0.025 | Small-to-moderate |
|  | Attitude | 8.183 | 6 | .225 | 0.007 | Negligible to small |
|  | Practice | 14.855 | 6 | .021 | 0.030 | Small |
| **Education Level in Pharmacy** | Knowledge | 6.616 | 3 | .085 | 0.012 | Small |
|  | Attitude | 6.962 | 3 | .073 | 0.013 | Small |
|  | Practice | 7.248 | 3 | .027 | 0.017 | Small |
